# Lessons learned from a lipid lowering trial in adolescents with type 1 diabetes

**DOI:** 10.1186/1687-9856-2012-24

**Published:** 2012-07-30

**Authors:** Franziska K Bishop, R Paul Wadwa, Sam Ellis, Marian Rewers, David M Maahs

**Affiliations:** 1 Barbara Davis Center for Childhood Diabetes, University of Colorado Denver, 1775 Aurora Ct., Mail Stop F527, Aurora, CO, 80045, USA

**Keywords:** Type 1 diabetes, Dyslipidemia, Pediatrics, Lipid-lowering medications

## Abstract

Herein, we describe recruitment efforts for a trial of lipid-lowering medications in adolescents with type 1 diabetes, age 12–21 years. Based on our experience, future studies will require multiple centers to enroll a sufficient number of participants for adequate data to direct dyslipidemia medication treatment guidelines for adolescents with type 1 diabetes.

## 

Adolescents and young adults with type 1 diabetes (T1D) are at high risk for developing early cardiovascular disease [1]. Current recommendations to consider pharmacologic treatment of elevated low-density lipoprotein cholesterol (LDL-c) are based on limited evidence and extrapolation from data in middle age and older adults. We proposed a trial of lipid-lowering medications (simvastatin, a statin, compared to Vytorin, a combination of simvastatin and ezetimibe, a medication that blocks cholesterol absorption) in our patients ages 12–21 years with LDL ≥ 130 mg/dl, consistent with current American Diabetes Association (ADA) guidelines. In this study, we hypothesized that simvastatin and Vytorin would be safe in adolescents with T1D and that in a two-arm design, Vytorin would lower LDL-c more than monotherapy with simvastatin at 6 months compared to baseline. In this report, we describe observations from a trial of lipid-lowering medications in T1D, age 12–21 years.

In 2004 we ascertained that the Barbara Davis Center for Childhood Diabetes (BDC) followed 1,528 patients with T1D age 12–21 years. The SEARCH for Diabetes in Youth study reported LDL-c >130 mg/dl in 15% of T1D subjects [2]. Based on these data, we estimated that 229 T1D patients age 12–21 years seen at the BDC would have LDL-c >130 mg/dl. A randomized, double-blind, placebo-controlled study of lipid-lowering medications required 82 enrolled subjects which assumed a 36% participation rate. Inclusion criteria were age 12–21 years with T1D diagnosed by positive islet autoantibodies or provider diagnosis of T1D, and LDL-c > 130 mg/dl. Patients with familial hypercholesterolemia, triglycerides > 400 mg/dl, T1D of less than three-months duration, HbA1c > 9.5%, abnormal thyroid function, abnormal creatine kinase values, abnormal liver function tests (ALT/AST), pregnancy or potential to become pregnant during the study, and patients on oral contraceptives were excluded.

## Findings

**Figure 1:**
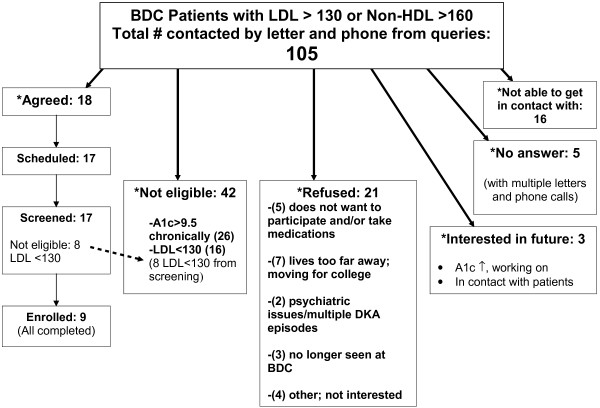
Study Recruitment and Participation.

We identified 105 potential subjects with clinically measured LDL-c >130 mg/dl or non-HDL-c >160 mg/dl. Of these, 42 patients proved to be ineligible (A1c > 9.5%, LDL-c < 130 mg/dl on a repeat test), 26 declined invitation to a screening visit, 16 expressed one time interest but were not able to be scheduled, and 3 were interested but were unable to improve their glycemic control to be eligible for the study. Therefore, 18 agreed to be in the study and 17 subjects attended a study screening visit of which 9 were enrolled in the study (15.8 ± 2.8 years, 67% male, A1c = 8.3 ± 1.1%, TC = 224 ± 42, HDL-c = 51 ± 11, TG = 112 ± 66, LDL-c = 151 ± 29 mg/dl, BMI = 25.4 ± 5.0 kg/m^2^) (Figure 1). Reasons for poor recruitment included elevated A1c (>9.5%), improved LDL-c from clinic lipid panels to screening visit (n = 42, 40%), lack of interest in taking a lipid-lowering medication and/or long distance to travel to study site (n = 21, 20%). A positive result of our study was improved compliance with lipid screening in our clinic population (a 55% reduction, from 416 to 188) in subjects eligible for, but lacking screening lipids over 2.5 years.

Despite current recommendations for intensified glycemic control, diet, and exercise as initial therapies for dyslipidemia, some adolescents with T1D will not reach the LDL-c goal of <100 mg/dl. For these patients, data are needed on the safety, efficacy, and ultimately the risk/benefit of dyslipidemia medications initiated in adolescence. We are aware of only one published short-term clinical trial of dyslipidemia medication in adolescents with T1D [3], although a Juvenile Diabetes Research Foundation sponsored trial is on-going in the United Kingdom [4]. Increased awareness of lipid health and treatment options in this patient population are needed as some patients and families required months of deliberation before deciding to have the patient take a dyslipidemia medication in addition to intensification of glycemic control, diet, and exercise. Based on our experience, future studies will require multiple centers to enroll a sufficient number of participants for adequate data to direct treatment guidelines for pharmacologic treatment of dyslipidemia in adolescents with T1D.

## Authors’ contributions

Author Contributions: FB, RPW, DMM researched data. FB wrote manuscript. FB, RPW, DMM, MR, SE reviewed/edited manuscript. FB, RPW, DMM, MR, SE contributed to discussion, reviewed/edited manuscript.
